# Abundance, Diversity and Role of ICEs and IMEs in the Adaptation of *Streptococcus salivarius* to the Environment

**DOI:** 10.3390/genes11090999

**Published:** 2020-08-26

**Authors:** Julie Lao, Gérard Guédon, Thomas Lacroix, Florence Charron-Bourgoin, Virginie Libante, Valentin Loux, Hélène Chiapello, Sophie Payot, Nathalie Leblond-Bourget

**Affiliations:** 1 Université de Lorraine, INRAE, DynAMic, F-54000 Nancy, France; julie.lao@univ-lorraine.fr (J.L.); gerard.guedon@univ-lorraine.fr (G.G.); florence.charron@univ-lorraine.fr (F.C.-B.); virginie.libante@univ-lorraine.fr (V.L.); sophie.payot-lacroix@inrae.fr (S.P.); 2 Université Paris-Saclay, INRAE, MaIAGE, 78350 Jouy-en-Josas, France; thomas.lacroix@inrae.fr (T.L.); valentin.loux@inrae.fr (V.L.); helene.chiapello@inrae.fr (H.C.)

**Keywords:** integrative and conjugative elements, integrative and mobilizable elements, conjugation, antibiotic resistance, metabolic functions

## Abstract

*Streptococcus salivarius* is a significant contributor to the human oral, pharyngeal and gut microbiomes that contribute to the maintenance of health. The high genomic diversity observed in this species is mainly caused by horizontal gene transfer. This work aimed to evaluate the contribution of integrative and conjugative elements (ICEs) and integrative and mobilizable elements (IMEs) in *S. salivarius* genome diversity. For this purpose, we performed an in-depth analysis of 75 genomes of *S. salivarius* and searched for signature genes of conjugative and mobilizable elements. This analysis led to the retrieval of 69 ICEs, 165 IMEs and many decayed elements showing their high prevalence in *S. salivarius* genomes. The identification of almost all ICE and IME boundaries allowed the identification of the genes in which these elements are inserted. Furthermore, the exhaustive analysis of the adaptation genes carried by these elements showed that they encode numerous functions such as resistance to stress, to antibiotics or to toxic compounds, and numerous enzymes involved in diverse cellular metabolic pathways. These data support the idea that not only ICEs but also IMEs and decayed elements play an important role in *S. salivarius* adaptation to the environment.

## 1. Introduction

Conjugation is a horizontal gene transfer (HGT) mechanism that massively contributes to the evolution of prokaryotic genomes [1,2,3]. It is mediated not only by extrachromosomal elements (i.e., plasmids), but also by other mobile genetic elements (MGEs) that are integrated into the chromosome or plasmids of their host (for a review [2]). Integrated elements that transfer by conjugation include: (i) the integrative and conjugative elements (ICEs), (ii) the integrative and mobilizable elements (IMEs) and (iii) decayed elements deriving from ICEs or IMEs, such as cis-mobilizable elements (CIMEs) (for reviews see [2,3,4]). ICEs are autonomous conjugative elements: they carry a recombination module and a conjugation module that together ensure the excision of the element, its transfer by conjugation, its replication during transfer and its integration in donor and recipient genomes (for reviews see [2,3,4,5,6]).

The ICE recombination module includes the genes and sequences dedicated to the excision from and integration into the bacterial chromosome or plasmid. It encodes one (or several) protein(s) belonging to one of the three phylogenetically and structurally unrelated families of enzymes: tyrosine integrases, serine integrases and DDE transposases [2,3,4,5,6,7]. Tyrosine and serine integrases generally catalyze excision by site-specific recombination between short direct repeats of the *att*L and *att*R flanking sites. This leads to an *attI* site that includes a single copy of this sequence. After transfer, most of them promote integration by catalyzing a site-specific recombination between this sequence from *attI* and another copy carried by the *attB* chromosomal site. As a consequence, the integrated ICE is flanked by DRs. The DDE transposases recognize terminal inverted repeats. The integration of elements encoding a DDE transposase generates target duplication, also leading to short DRs flanking the element.

The conjugation module of all ICEs from Firmicutes is dedicated to their transfer as single-strand DNA. It encodes a relaxase, a coupling protein (CP) and a “type IV secretion system” (T4SS) [8,9], including VirB4, a conserved ATPase providing energy [10]. Three distinct superfamilies of relaxases have been identified thus far in ICEs from Firmicutes: the MobP, MobC and MobT [2,11,12]. The relaxase recognizes and cleaves one of the DNA strands of the circular element, specifically at the *oriT* site [13,14,15]. It is then recognized by a membrane-associated CP belonging to either the VirD4 or TcpA superfamilies. Together, CP and T4SS ensure the translocation of the relaxase-tethered DNA from the donor to the recipient bacteria. A rolling-circle replication of the element is likely concomitant to its transfer, so that the ICE is not lost in the donor cell. Finally, the relaxase achieves the transfer by recircularizing the ICE (for a review see [2]).

IMEs are mobile elements that dispose of a recombination module similar to that of ICEs that allows their autonomous integration and excision. However, they cannot self-transfer. Instead of a conjugation module, they carry a mobilization module that does not result from a recent decay of a conjugation module. It ensures their mobilization in trans: IMEs subvert the conjugative machinery of a co-resident conjugative element (plasmid or ICE) to promote their own transfer [3]. Known IMEs use many different mobilization strategies and therefore exhibit diverse mobilization modules [3]. In this work, we consider as IMEs only elements whose mobilization module encodes a relaxase, eventually a CP but no T4SS protein (no VirB4). In Firmicutes, IMEs encode relaxases that belong to superfamilies found in ICEs (MobP, MobC or MobT) and conjugative plasmids (MobV or MobQ). We recently proposed that IMEs could also encode relaxases belonging to superfamilies of initiators of rolling circle replication harboring PF01719, PF01719-PF00910, PF02407 or PHA00330 domains [16].

CIMEs are decayed elements deriving from ICEs and IMEs by deletion that retained their *att* recombination sites but not their conjugation/mobilization modules and all their genes involved in recombination (for a review see [2]). Hence, CIMEs can only transfer by cis-mobilization resulting from an accretion-mobilization process [17]. This latter takes place when an ICE or an IME integrates in one of the attachment sites flanking the element resulting in a composite element that can excise and transfer by conjugation.

The vast majority of these integrated mobile elements carry adaptive genes that may confer on their host a significant selective advantage or may change their lifestyle (e.g., antibiotic, heavy metal or phage resistance, sucrose catabolism, bacteriocin synthesis, pathogenicity or symbiosis) [2,3,10,18,19,20,21]. It is therefore important to study the prevalence of these elements and to identify the adaptive function they tend to disseminate among bacterial populations.

In this work, we focused on *S. salivarius*, a species of Firmicutes that belongs to microbiomes of all humans and contributes to the maintenance of oral, pharyngeal and gut health [22,23,24]. Some *S. salivarius* strains are also described as opportunistic pathogens since they have been associated with cases of meningitis [25,26], endocarditis [27] and bacteremia in immunocompromised patients [28,29]. *S. salivarius* genomes are known to evolve rapidly, presumably through HGT [30]. Indeed, there is accumulating evidence of the pivotal role played by conjugative and/or mobilizable elements in *S. salivarius* HGT [31]. In a previous study, we highlighted the occurrence of ICEs belonging to the ICE*St3* family in 13 *S. salivarius* genomes [32]. In this work, we enlarged the number of *S. salivarius* genomes analyzed (*n* = 75) and searched not only for ICE*St3* elements but also made an exhaustive search of the diversity and abundance of all ICE and IME families. We delimited almost all the identified elements, precisely defined their insertion site and searched for CIMEs that are integrated in tandem with ICEs and IMEs. We also characterized the adaptive functions carried by these mobile elements. Altogether, these data shed light on the diversity and prevalence of ICEs and IMEs in *S. salivarius*. They also make a comprehensive picture of the role of these mobile elements in *S. salivarius* adaptation to the environment.

## 2. Materials and Methods

### 2.1. S. salivarius Genomes and Phylogenetic Analysis

#### 2.1.1. *S. salivarius* Strains and Genome Analysis

In this work, the genomes of 75 *S. salivarius* strains were analyzed. Dates and sites of sampling of the strains are given in Table S1. Twenty of the 21 strains isolated from our strain collection [33] were subjected to whole genome sequencing using an Illumina HiSeq2000 sequencer by Beckman Coulter Genomics (2 × 100 bp after paired-end library construction, at least 60 × coverage). De novo assemblies were performed using CLC Genomics Workbench (CLC Bio) using default parameters. Scaffold of the genomes was built by using the Genome Finishing module of CLC Genomics Workbench with the *S. salivarius* JIM8777 genome as Guédon et al. [34]. Some assembly gaps were filled by PCR and sequencing. Automatic annotation for each genome utilized the pipeline AGMIAL (Bryson 2006 agmial). The sequences (raw reads and assembled scaffolds) of the 20 strains have been deposited in the EBI-ENA database under the study number PRJEB37543. Accession numbers of the assemblies are also indicated in Table S1. The coordinates of the elements are included in Table S2. The remaining 54 genomes were retrieved from the NCBI genome databank either as complete genomes (*n* = 7) or as scaffolds of WGS (*n* = 47) (last accessed may 2019). Pseudocontigs were generated using CONTIGuator (http://combo.dbe.unifi.it/contiguator, with default parameters) with the *S. salivarius* JIM8777 genome as reference. Unmapped contigs were added at the end of the pseudocontigs. When available, the annotations were transferred to contigs using Geneious prime 2020.1.1. The nucleotidic sequences of elements are available in the Supplementary File S1—55.

#### 2.1.2. Phylogenetic Tree Based on Single Nucleotide Polymorphisms (SNPs)

Phylogenetic relationships among *S. salivarius* strains were evaluated by analysis of single nucleotide polymorphisms (SNPs) using the CSI Phylogeny software (version 1.4) on the CGE website (https://cge.cbs.dtu.dk/services/CSIPhylogeny/). A multifasta file was generated using the NCTC8618 type strain as reference and including SNPs of all the 75 *S. salivarius* aligned genome assemblies. The phylogenetic tree of the strains was inferred using the Maximum Likelihood method based on the Tamura-Nei model implemented in the Mega7 software [35].

### 2.2. Detection and Delineation of the Integrative Elements

The workflow used to detect, delineate and classify ICEs was described previously [12]. Briefly, for ICE identification, signature proteins of the recombination module (integrase) and the conjugation module (relaxase, CP and VirB4) were searched by BLASTp comparison (BLAST 2.9.0+) against a curated database of 1029 signature proteins extracted from Firmicutes ICEs and IMEs. False positives were then filtered out by retaining only candidates that meet the four following criteria: the identity percentage (≥25%), the alignment coverage (≥40%, must cover the functional domain), the *E*-value (≤1 × 10^−5^ for CP and VirB4, ≤1 × 10^−4^ for relaxase and integrase) and the hit length (≥320 aa for integrase, ≥180 aa for relaxase, ≥500 aa for VirB4, between 180 aa and 700 aa for short CP and between 1000 aa and 1200 aa for long CP). Genes encoding signature proteins were then co-localized. If all four proteins were present, the element was considered as ICE. Its delineation was done by searching for DRs at their two ends by BLASTn analysis (word = 7 bp) using either the 3′ or the 5′ end of the potential target gene as query. When DRs were absent, too short or too degenerated to be detected by BLASTn, the sequence of the region containing signature proteins was compared to chromosomal sequences of *S. salivarius* strains devoid of the analyzed element and/or to sequences of elements sharing a very closely integrase using Megablast (word = 16 bp). The DRs were then identified by manually comparison of the ends of syntenic regions. To resume, all elements flanked by recombination sites and/or DRs and which encode an integrase, a relaxase, a CP and a VirB4 were considered as ICEs. Elements that lack one or two of these characteristics and clearly derive from a closely related ICE were counted as dICEs (decayed ICEs). The classification of ICEs/dICEs into families was based on the nature of the proteins of the conjugation module and was carried out as previously described [12].

IME identification and delineation were done as previously defined [16]. Briefly, IME detection is based on the combined presence of a recombination module (detected by its integrase) and a mobilization module (dedicated relaxase, eventually a CP and absence of other proteins of the conjugation module). The detection of genes encoding these signature proteins and the filtration of false positives were performed as described above. Even if various genuine IMEs from proteobacteria and one of firmicutes do not encode their own relaxase [3], elements were considered as IMEs in this work if: (i) they encode their own putative relaxase, regardless of their ability to encode a putative CP, (ii) they do not carry any putative VirB4 gene or pseudogene and (iii) their relaxase (and eventually their CP) is very distantly related (<40% identity) or unrelated to any relaxase (or CP) of any ICE or conjugative plasmid. Elements were identified as dIMEs (decayed IMEs) if a signature protein or *att* site was predicted non-functional. Integrated elements that encode their own integrase but do not encode other signature proteins (nor prophage signature genes) were counted as mobile genomic islands (MGIs). Decayed elements were counted as CIMEs if they were found devoid of functional genes encoding all the signature proteins. The method used in this work (search of signature genes and flanking recombination sites/DRs) allows the detection of CIMEs only if they are integrated in tandem with ICEs, dICEs or IMEs. As a reminder, the Figure S1 schematizes of the attributes used to discriminate the different types of elements.

To evaluate the number of these elements, we chose to count separately the elements that appear integrated in tandem, whatever the nature of the element. The denomination of the elements includes the putative nature of the element (ICE, IME, CIME or MGI), the host strain and its insertion site or specificity, for example IME_*SsalL25*_*oriT*.

Circos6 [36] was used to show associations of signature proteins in elements and the content of MGEs of each strain. Manual editing of the figures was done using Inkscape.

### 2.3. Characterization of Cargo Genes Encoded by ICEs and IMEs

Taking into account data on cargo genes in the literature, 31 functional categories related to fitness and 10 category tags were defined. The attribution of cargo genes encoded by ICEs and IMEs to these categories was based on keywords found in the functional annotation of the genes or on alignment with six external resources with significant hits: AMRFinderPlus [37], BACTIBASE [38], VFDB [39], REBASE [40], NORINE [41] and MEROPS [42]. AMRFinderPlus is packaged with its own search engine (AMRfinder) and was used with default parameters. BLASTp was used to query the other resources. Alignments were considered significant if they met the following stringent criteria: (i) for protein of size ≤ 100 aa: coverage ≥ 80%, identity ≥ 80%, e-value ≤ 1 × 10^−10^; for protein of size > 100 aa and ≤250 aa: coverage ≥ 70%, identity ≥ 70%, e-value ≤ 1 × 10^−20^; (ii) for protein of size > 250 aa and ≤500 aa: coverage ≥ 65%, identity ≥ 65%, e-value ≤ 1 × 10^−40^; (iii) for protein of size > 500 aa: coverage ≥ 60%, identity ≥ 60%, e-value ≤ 1 × 10^−60^. The assignments in the categories were carried out manually.

## 3. Results

### 3.1. ICEs in S. salivarius Genomes

#### 3.1.1. ICE Prevalence and Diversity

**Figure 1:**
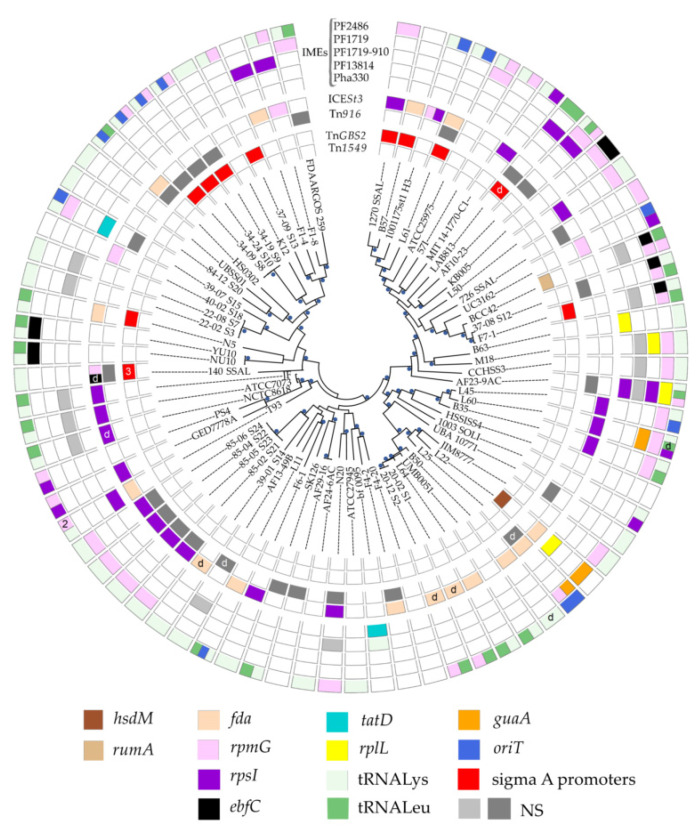
Integrative and conjugative elements (ICEs) and integrative and mobilizable elements (IMEs) carried by *Streptococcus salivarius* genomes. Strains are indicated in the middle of the circle and were grouped according to their phylogenetic relatedness. Groupings with bootstrap values >90 are marked with blue dots. Boxes located in front of a strain name are either empty (absence of element) or colored (presence of an ICE or an IME). The different families of ICEs and IMEs are indicated at the top in the opening of the circle. The first four inner circles indicate the presence of elements belonging to the four distinct families of ICEs retrieved in this study. The five outer circles show the presence of IMEs, where each line corresponds to one IME superfamily. Defective ICEs or IMEs are indicated by a “d.” The box is split in two (or more) when two elements (or more) of the same superfamily are present at distinct integration sites. If several elements of the same superfamily are present at the same integration site, the number of elements is indicated in the box (for example, two IMEs with a PF02486 domain in strain T93). The colored boxes indicate the genes where the elements are inserted. The medium-grey boxes materialize low-specific integration (NS).

Among the 75 *S. salivarius* genomes analyzed, more than 2/3 (*n* = 53) carry at least one ICE or dICE and only 22 are devoid of ICEs Table S3, Figure 1. A total of 69 ICEs (and eight dICEs) were identified and classified into superfamilies and families. This classification was carried out as previously described [13] and takes into account the nature of the domains carried by signature proteins for superfamilies (relaxase, VirB4 and CP) and the 40% identity clustering of these signature proteins for families. Among the seven families of ICEs described thus far in streptococci [12], four are present in the genomes of *S. salivarius*: Tn*916*, ICE*St3*, Tn*GBS2* and Tn*1549* families.

In a previous work, we already explored the presence of ICE*St3*-related elements for 13 of the 21 strains of our collection [32]. For the sake of completeness, we show here all the elements present in these strains Table S3, Figure 1. ICE*St3*-related elements are not only present in 18 of the 21 strain of our collection but also in other *S. salivarius* genomes. Thus, this family of ICEs is the most prevalent one (*n* = 38 including five dICEs). Two other families of ICEs were also found in abundance: Tn*916* family (*n* = 24 including two dICEs) and Tn*GBS2* family (*n* = 13 including one dICE). Two Tn*916* (including one dICE) were found on megaplasmids. Only two strains carry a Tn*1549*-related element.

Most genomes (*n* = 34) exhibit only one ICE or dICE, but 18 genomes carry several ICEs and/or dICEs, usually two that belong to two distinct ICE families Figure 1. The most frequent co-occurrence is Tn*916*-related element with ICE*St3*-related element (*n* = 10). Only two strains encode two ICEs belonging to the same family (strains 140 SSAL and 1001175st1_H3 with two ICE*St3*-related elements). Strain 140 SSAL differentiates from other strains by its richness in ICEs since it harbors six ICEs or dICEs belonging to Tn*916* (*n* = 1), ICE*St3* (*n* = 2 including one dICE) and Tn*GBS2* (*n* = 3) families. 

Most of the Tn*916*-related dICEs or ICEs harbor regulation, conjugation and integration modules are almost identical to those of Tn*916* (>99% identity). Many of these elements carry some transposon insertion(s), in particular in *orf*9, that were previously identified in closely related ICEs [43]. However, two very closely related ICEs (ICE_*SsalL61_Tn916* and ICE_*SsalLAB813_Tn916* showing >99% identity), which do not carry any transposon, share only 90% identity with Tn*916* over their entire length. Two other elements seem to be chimerical. Thus, the left part of dICE_*SsalAF13-49B_Tn916* (from *attL* to an internal position of *orf16*) is almost identical to ICE_*SsalLAB813_Tn916*, whereas its right part is almost identical to that of Tn*916* (>99% identity) with a transposon insertion in *orf*9. This suggests that this element is a chimera resulting from a homeologous recombination between two Tn*916*-related elements sharing only 90% identity. Furthermore, the left part of ICE_*SsalSK126_Tn916* (from *attL* to the *nic* site cut by the relaxase) shares only 91% identity with Tn*916*, whereas its right part is almost identical to Tn*916* (>99% identity) with an insertion in *orf*9. This suggests that this element is a chimera resulting from a recombination catalyzed by the relaxase of the element between two Tn*916*-related elements sharing only 91% identity. Such recombinations were previously reported for other ICEs but not for Tn*916*-related elements (for a review see [2]).

#### 3.1.2. ICE Integrases and Integration Sites

In ICEs/dICEs found in *S. salivarius*, the most prevalent integrases are tyrosine integrases (*n* = 62) that are found in 80% of them. As expected, the tyrosine integrase encoded by the elements of the Tn*916* family has a low specificity of integration. All ICE*St3*-related elements (including dICE) encode a tyrosine integrase and are integrated in four well-conserved housekeeping genes of *S. salivarius* genomes, i.e., in the 3′ end of *rpsI* (encoding the S9 ribosomal protein) (*n* = 17 including one dICE), *fda* (fructose-1,6-bisphosphate aldolase gene) (*n* = 15 including three dICEs) and *rpmG* (encoding the L33 ribosomal protein) (*n* = 5) and in the 5′ end of *ebfC* (encoding a nucleoid associated protein) (*n* = 1 dICE) Table S3, Figure 1.

The second most prevalent integrases in ICEs/dICEs of *S. salivarius* are the DDE transposases belonging to the IS*Lre2* transposase family. These integrases were exclusively found in ICEs belonging to the Tn*GBS2* family (*n* = 13 including 1 dICE) Table S3, Figure 1. DDE transposases of this family target diverse sigma A promoters and therefore avoid integration of elements into various genes [44,45]. Our results are consistent with these findings.

The two ICEs belonging to the Tn*1549* family rely on serine integrase(s) for their integration/excision. They are specific of sites located within some widespread but dispensable genes. One ICE encodes a serine integrase that targets *rumA* (encoding a 23S rRNA (uracil-5-) methyltransferase). The other encodes a triplet of serine integrases in the same orientation and is inserted in *hsdM* (encoding a methyltransferase subunit of a type I restriction-modification system) Table S3, Figure 1.

#### 3.1.3. Slightly Decayed Elements Deriving from ICEs

Eight dICEs which are closely related to ICEs belonging to the ICE*St3*, Tn*916* or Tn*GBS2* families were found. Four of them result from the pseudogenization of one of the genes encoding a signature protein and another cannot be precisely delimited but seems to lack relaxase and CP encoding genes.

Two strains, Ssal20-12 S2 and Ssal20-02 S1, carry ICE*St3*-related identical dICEs integrated into the 3′ end of their *fda* gene. In ICE*St3*, the conjugation and recombination modules are transcribed as a unique operon (*orfONMLK-oriT-orfJIHGFEDBA-xis-int*) [46]. The related region of both dICEs differs by a large but precise internal deletion of *orfIHGFEDBA* that probably encodes the T4SS. These dICEs contain all other genes resulting in a putative operon *orfONMLK-oriT-orfJ-xis-int*, which shares 94.1% identity with the related sequences of ICE*St3*. Another element, dICE *Ssal39-01*_*fda,* has also this large deletion of *orfIHGFEDBA* (located exactly at the same position as the one found in dICE*_Ssal20-12_fda*). However, it is different from the other two since it has two additional deletions in *orfK* (one including the 5′ end of the gene and another within the gene). Its *orfONMLψorfK-oriT-orfJ-xis-int* putative operon shares only 94.2% identity with the related region of dICE*_Ssal20-12_fda.* These three elements could be derived from ICE*St3*related elements by deletion and can no longer transfer autonomously. They could be mobilizable by using the functional T4SS of ICE*St3*-related ICE.

### 3.2. IMEs in S. salivarius Genomes

#### 3.2.1. IME Prevalence and Diversity

Among the 75 *S. salivarius* genomes, more than 90% (*n* = 69) carry at least one IME/dIME and only six genomes are devoid of it. Among the latter, two are also devoid of ICEs (*S. salivarius* HSISS4 and *S. salivarius* F4-20). A total of 165 IMEs and two dIMEs were identified, one of which is carried by a plasmid (IME_*SsalM18_rpmG*) (Table S3). As seen in Table S3, the occurrence of IMEs/dIMEs varied within strains. Numerous chromosomes carry two IMEs/dIMEs (*n* = 30) but this number can go up to seven in strain AF23-9AC.

These 167 IMEs/dIMEs were classified into five superfamilies in Table 1 by taking into account the domain composition of their relaxase that is the main protein of the mobilization module. The most prevalent superfamily of IMEs/dIMEs is the IME_PF02486 superfamily (*n* = 113, including two dIMEs) that comprises elements with a relaxase exhibiting a PF02486 domain Table 1, also known as Rep_trans. The four other superfamilies of *S. salivarius* IMEs/dIMEs are IME_PF01719 (*n* = 28), IME_PF13814 (*n* = 13), IME_PHA00330 (*n* = 6) and IME_PF01719-PF00910 (*n* = 7).

The 40% sequence identity clustering of the relaxases sharing the same catalytic domain allows to classify relaxase families. In total, 11 families of relaxases were retrieved Table 1, column 2. The comparison of these families with those previously described in streptococci [16] reveals the existence of a new family of relaxases, Rel_PF01719-PF0910_5.

In this previous study [15], we also found that half of the streptococcal IME mobilization modules include a CP and that all these CPs except two do not belong to the canonical VirD4 superfamily but to the TcpA superfamily. Here, the percentage of *S. salivarius* IMEs/dIMEs encoding a CP is somewhat lower (33%, *n* = 54) and the majority of these CPs also belong to the TcpA superfamily, but the fraction is somewhat lower (77%, *n* = 42). A 40% sequence identity clustering of CP allows to subdivide the TcpA superfamily into seven families, see Table 1, column 3. The TcpA_2, TcpA_6 and TcpA_12 families were already described [15], whereas TcpA_13, TcpA_14 and TcpA_15 are novel families. The most prevalent family is the TcpA_12 (*n* = 32), as previously described for streptococcal IMEs [16].

The VirD4 proteins identified in this work (*n* = 12) all belong to the IME_PF13814 family. The association of a VirD4 protein with a Rel_PF13814 was already described in IME mobilization modules [16]. However, one Rel_PF13814 is not associated with a CP, which is unusual.

#### 3.2.2. IME Integrases and Integration Sites

*S. salivarius* IMEs/dIMEs encode integrases belonging to two unrelated superfamilies of recombinases, tyrosine integrases and serine integrases. Serine integrases were found in 12 IMEs that all belong to the IME_PF13814 superfamily Table 1. All these elements are integrated in intergenic regions, suggesting that these integrases are specific of elements or structures found in promoters or terminators, as the DDE transposases encoded by ICEs belonging to Tn*GBS1* and Tn*GBS2* families.

Tyrosine integrases were detected in more than 92% of the IMEs (*n* = 155/167). Most of the tyrosine integrases (*n* = 133) specifically target the 3′ end of housekeeping genes such as genes encoding tRNAs (tRNALys *n* = 55; tRNALeu *n* = 22) or ribosomal proteins being either *rpsI* (*n* = 13), *rpmG* (*n* = 39) or *rplL* (*n* = 4). More rarely, tyrosine integrases from IMEs catalyze integration at the 3′ or 5′ end of other protein-encoding genes *(guaA* (*n* = 3), *tatD* (*n* = 2) or *ebfC* (*n* = 6)). The last 11 IMEs encoding closely related tyrosine integrases are specifically integrated in the *oriT* sequence of ICEs belonging to the ICE*St3* (*n* = 6) or Tn*916* (*n* = 1) families or are integrated in secondary sites (*n* = 4) (see [47] for more details).

#### 3.2.3. Diversity of Integrase-Relaxase-CP Combinations within IMEs

**Figure 2:**
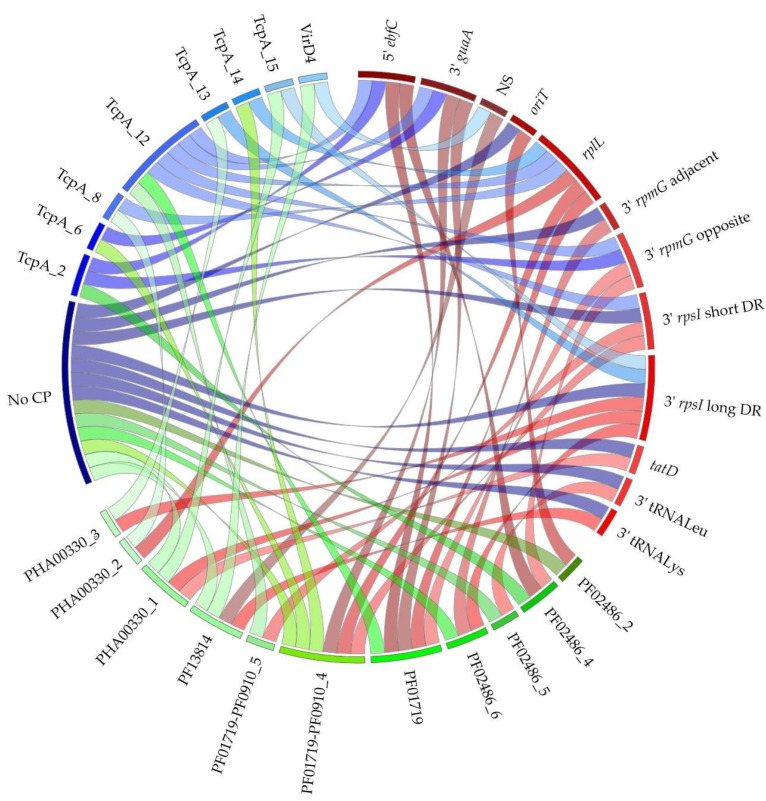
Diversity of integrase-relaxase-CP combinations for IMEs/dIMEs. Arcs show the group proteins belonging to the same family according to phylogenetic analysis and 40% sequence identity clustering: red, green and blue arcs for clustered integrases, relaxases and CPs, respectively. Ribbons indicate the association between integrases and relaxases in red, relaxases and CPs in green and CPs and integrases in blue.

The analysis of the co-occurrence of the superfamilies and specificity of integrases, families of CPs and families of relaxases did not reveal exclusive associations, see Table 1 and Figure 2. Altogether, 21 different ternary associations were observed, suggesting a high frequency of shuffling between signature proteins. Among these associations, five have never been observed before: those involving the three newly identified superfamilies of CPs (TcpA_13; TcpA_14 and TcpA_15), those involving the newly discovered Rel_PF01719-PF0910_5 family of relaxases associated with CPs of the TcpA_12 family and those from the IME_PF13814 superfamily that are composed of a Rel_PF13814 associated with a tyrosine integrase in the absence of CP.

### 3.3. Composite Elements in S. salivarius Genomes

In the 75 analyzed genomes, we found 33 complex genomic islands consisting of two elements integrated in tandem and four genomic islands composed of three elements. These composite genomic islands are integrated in the 3′ end of *rpmG* (*n* = 12), *rpsI* (*n* = 16), *fda* (*n* = 8) and *rplL* (*n* = 1).

CIMEs found in these structures can be divided in two classes according to their size and gene content: (i) classical CIMEs which carry cargo genes and have a size from 1.2 to 13 kb and (ii) microCIMEs, which do not carry any gene and have a size from 130 to 220 bp.

The 37 composite structures include 16 ICEs, three dICEs, 27 IMEs, 19 classical CIMEs and 11 microCIMEs. One of these composite structures include another mobile genetic element, MGI*_SsaL22_rplL*, which encodes its own tyrosine integrase, but does not carry any gene (or pseudogene) encoding relaxase, CP or VirB4. The analysis of this large element (32.3 kb) suggests that it does not derive from any ICE or IME. We found single related large elements (28–38 kb) with similar features in six other strains, suggesting that they are not decayed elements.

The order and the nature of the elements in these 37 composite structures resulting from accretion are highly variable. Table 2 indicates the different combinations of the tandem elements relative to the position of the target gene. Several rules seem to emerge from this analysis: (i) two ICEs are never integrated in tandem, (ii) two IMEs belonging to the same superfamily are never integrated in tandem, (iii) the most decayed elements, i.e., CIMEs and microCIMEs, are always the most distant from the target genes.

Complex elements can also result from the integration of an element within another, resulting in a matryoshka element. Ten matryoshka elements were identified: an ICE carrying an IME integrated in its *oriT* (see [47] for further details) (*n* = 7) and conjugative plasmids carrying a Tn*916*-related ICE (*n* = 1) or a Tn*916*-related dICE (*n* = 1), or an IME (*n* = 1).

### 3.4. Function Encoded by the Cargo Genes

**Figure 3:**
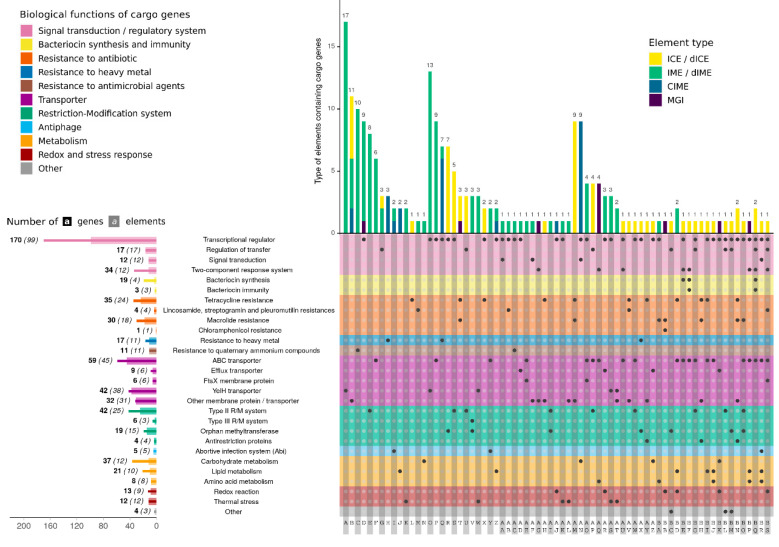
Function of cargo genes encoded by ICEs, IMEs and other MGEs from *S. salivarius*. The upper left indicates the different biological functions of the cargo genes. The upper right indicates the type of elements carrying the cargo genes. The lower left indicates the nature of cargo genes, their number (in bold) and the number of elements that encodes them (in brackets). The lower right connects the cargo functions with the different types of elements among them, IME_*Ssal1003-SOLI_rpsI* carries a cluster of four genes probably involved in the synthesis of polyketide fatty acid and ICE_*SsalL11_fda* displays a cluster of six contiguous genes that are potentially involved in the synthesis of fatty acids. Furthermore, two ICEs (ICE_*SsalF6-1_rpsI* and ICE_*SsalL25_fda*), one IME (IME_*SsalAF10-23_tRNAleu*) and five MGIs each encode one protein that potentially functions as an asparagine synthetase (*n* = 5), a 2-amino-3-ketobutyrate coenzyme A ligase (*n* = 2) or an aminotransferase (*n* = 1) that could potentially be involved in amino acid metabolism.

In addition to functions that are essential for their mobility, almost all conjugative and mobilizable elements carry cargo genes, i.e., genes not involved in the transfer of the element. In this work, blast analysis against diverse databases (AMRFinderPlus, BACTIBASE, VFDB, REBASE, NORINE and MEROPS) were undertaken to identify cargo genes encoded by *S. salivarius* elements. This allowed assigning the biological function of 667 potential cargo genes Figure 3. Figure S2 lists the elements carrying cargo genes and indicates their presumed function. The most frequent function was the one corresponding to “Signal transduction and regulatory system” that includes 230 genes of which 168 encode transcriptional regulators. This number is overestimated since it is not possible to precisely distinguish cargo regulatory genes from those dedicated to the control of ICEs and IMEs transfer. Nevertheless, many of these transcriptional regulators can be considered as cargo genes such as the genes found in seven *S. salivarius* elements that encode proteins homologous to the DeoR/GlpR-type known as a regulator of sugar metabolism [48]. These genes are all located next to a cluster of genes involved in carbohydrate metabolism in accordance with their presumed function. Another example is the *cadX*-like genes found in four elements that likely act as cadmium transcriptional repressors [49] of a gene cluster located nearby and that likely confers resistance on Cd^2+^ and Zn^2+^.

Cargo regulatory genes also include 34 genes encoding transcriptional regulators and kinases probably composing two component systems (TCSs). These allow bacteria to sense and respond to changes in their environment [50]. They are carried by 12 *S. salivarius* elements. Five of the kinases carried by MGIs are related to ComD, a protein that leads to the activation of the competence regulon [51]. Eight CIMEs encode proteins with PAS/PAC domains that are commonly involved in environmental sensing (presumably of oxygen, redox, light or metals) [52,53].

Transporters is the second class of cargo genes (*n* = 148). This class comprises ABC transporters (*n* = 59) that couple the energy stored in adenosine triphosphate (ATP) to the movement of molecules across the membrane [41]. Six of them likely belong to the major facilitator superfamily (MFS) of transporters that are involved in the transport of a variety of substrates including antibiotics. Other transporters homologous to FtsX-like permeases (*n* = 6) or characterized by a YeiH domain (COG2855) of unknown function (*n* = 42) were retrieved. Diverse other membrane proteins (*n* = 32) were also identified but we were not able to assign them a precise function.

The third most frequent function encoded by cargo genes corresponds to restriction/modification (RM) systems. A total of 67 genes encoding restriction or modification proteins were retrieved. These include 42 type II RM proteins that are found on ICEs and IMEs. Type III RM (*n* = 6) are exclusively present on IMEs. Additional orphan methyltransferases (*n* = 19) were also retrieved frequently on ICEs or IMEs.

*S. salivarius* conjugative and mobilizable elements also encode proteins conferring antimicrobial resistance to their host cell. These include QacE efflux transporters (*n* = 11) that are carried by eight IMEs and likely confer resistance to quaternary ammonium compounds (QACs). They also comprise 70 proteins conferring resistance to diverse antibiotics. Indeed, 35 cargo genes were homologous to genes involved in resistance to tetracycline (*tet*(M)), 30 to macrolides (*erm*(B), *erm*(C), *erm*(F), *mef*(A), *msr*(D)), four to lincosamines (*lsa*(C)) and one to chloramphenicol (*catA*). At least 22 mobile elements of *S. salivarius* encode genes involved in resistance to antibiotics. The tetracycline and erythromycin resistance genes were mostly found on Tn*916*-related elements (*n* = 14). Four elements, two ICEs and two IMEs integrated in *oriT* of conjugative elements (IME_*SsaHS0302_oriT* and IME_*Ssa1001175st1_H3_oriT*, see [47]), encode a *lsa*(C) gene that is known to confer cross-resistance to lincosamides, streptogramin A and pleuromutilins in *S. agalactiae* [54]. Lastly, one MGI (MGI_*SsaF4-20_rumA*) encodes a chloramphenicol acetyltransferase that confers resistance to chloramphenicol. In addition to antibiotic resistance genes, 21 genes were found to be likely involved in bacteriocin synthesis and immunity. These were found on three ICEs and one dICE Figure S1. In addition, genes involved in resistance to the cadmium heavy metal (*n* = 17) were quite well represented in *S. salivarius* elements, mostly within CIMEs.

Elements also encode many proteins (*n* = 64) that possibly play a role in the cellular metabolism Figure S2. Indeed, 14 elements, mostly CIMEs (*n* = 10), each encode one to three proteins homologous to enzymes involved in carbohydrate metabolism. These enzymes potentially catalyze diverse functions such as 1-phosphofructokinase (*n* = 8), galactose-6-P isomerase (*n* = 4), tagatose-2P aldolase (*n* = 2), UDP-N-acetylglucosamine 2-epimerase (*n* = 7) and glycosyl transferase (*n* = 13). Several of these enzymes encoded by an MGI in the strain *S. salivarius* SK12 are likely responsible for the lactose assimilation via the tagatose-6 phosphate pathway [37]. In addition, 21 other cargo proteins are homologous to proteins involved in lipid metabolism. They are likely involved in diverse functions: synthase (e.g., 3-oxoacyl-[acyl-carrier-protein] synthase (*n* = 5), β-ketoacyl synthase (*n* = 1)), reductase (e.g., 3-oxoacyl-[acyl-carrier-protein] reductase (*n* = 1)) or kinase (diacylglycerol kinase (*n* = 3)). These enzymes are encoded by 10 elements (four ICEs, two IMEs, one MGI and three CIMEs). Lastly, *S. salivarius* integrative elements also encoded proteins that are required for stress tolerance. Indeed, nine elements (two CIMES, five IMEs and two ICEs) encode proteins (*n* = 13) that could catalyze redox reactions. Twelve other elements harbor one gene encoding a protein homologue to the LtrA protein which has been found to be essential for growth at low temperature (4 °C) in *Listeria monocytogenes* [55].

## 4. Discussion

Although ICEs belonging to the ICE*St3* and Tn*916* families were previously reported in some strains of *S. salivarius* [12,32,33], the real prevalence and diversity of ICEs in this species was still unknown. The present analysis identified 69 ICEs and eight slightly decayed ICEs (dICEs) in the 75 analyzed genomes, revealing a high prevalence of ICEs in this species. All these elements belong to the superfamilies (based on the domain content of their relaxases and CPs) and families (based on identities >40% of relaxases, CPs and VirB4) previously identified in the *Streptococcus* genus [12]. Most of them belong to the two previously reported families in *S. salivarius*, ICE*St3* (*n* = 38) and Tn*916* (*n* = 24), that encode MobT relaxases, TcpA CPs, MPF_FA_ T4SSs and tyrosine integrases. One of these ICE*St3* elements is specifically integrated in the 5′ end of *ebfC,* an integration site that has not yet been identified in *S. salivarius*. Among the *S. salivarius* set of genomes, 13 have been initially screened and selected because they carry members of the ICE*St3* family [32]. However, ICE*St3*-related elements are also present in many other genomes (25 ICEs or dICEs in 62 strains), showing the high prevalence of this ICE family in this species. The complete genomes retrieved from Genbank that were analyzed in this study include many strains that have been initially sequenced and studied because they are resistant to antibiotics. Therefore, since Tn*916*-related ICEs from streptococci confer resistance to antibiotics, the high frequency of the Tn*916* family found in this work is probably overestimated. Our analysis also revealed ICEs or dICEs belonging to two other families, Tn*GBS2* family (*n* = 13) and Tn*1549* family (*n* = 2), that have not been previously reported in *S. salivarius*. These two distantly related families encode MobP relaxases, VirD4 CPs and MPF_FATA_ T4SSs. Their DDE transposases or serine integrases have specificities that have never been reported in *S. salivarius*: (i) sigma A promoters for DDE transposases of Tn*GBS2*-related ICEs and (ii) internal sites of *rumA* or *hsdM* for serine integrases of Tn*1549*-related ICEs. Overall, this study extends the repertoire of ICEs and of their integration sites in *S. salivarius.*

An initial study of 1124 prokaryotic genomes based on the chromosomal location of complete conjugation modules (probably carried by ICEs) or of relaxase genes devoid of accompanying T4SS genes (probably carried by IMEs) suggested that IMEs slightly outnumber ICEs [1]. Thereafter, this prediction was corroborated by the only exhaustive searches of ICEs and IMEs encoding a relaxase in a large amount of strains: on 124 genomes of various streptococci [12,13,14,15,16] and on 214 genomes of *Streptococcus suis* [18]. Unexpectedly, our exhaustive search of IMEs in a large set of *S. salivarius* revealed many more IMEs (*n* = 165) than ICEs/dICEs (*n* = 77). It should also be emphasized that many IMEs from proteobacteria and a few from Firmicutes carry their own integrase gene and *oriT* but do not encode any relaxase. In this study, elements encoding their own integrase and devoid of relaxase were not considered as IMEs but as MGIs. Therefore, the high prevalence of IMEs that we have found in *S. salivarius* could be underestimated.

This work revealed not only a large number of IMEs but also a huge diversity of their: (i) integration specificities (10 different integration specificities compared to seven for ICEs), (ii) relaxases (five superfamilies and 11 families compared to two superfamilies and four families for ICEs), and (iii) CPs (two superfamilies and eight families compared to only two superfamilies and four families for ICEs). Some families (three families of CPs and one family of relaxases) are reported here for the first time. In total, IMEs exhibit 21 different combinations of integrase-relaxase-CP families and insertion specificities Table S3 compared to seven for ICEs. Overall, this study greatly extends the repertoire of IMEs in *S. salivarius*.

This work is the first exhaustive search of *cis*-mobilizable elements (CIMEs) integrated in tandem with ICEs and IMEs in a large set of strains. It revealed 32 CIMEs or microCIMEs. However, our method did not allow to identify single CIMEs, and therefore probably missed many elements. The amount of detected CIMEs (*n* = 30) is similar to that of IMEs integrated in tandem and largely outnumbers the amount of ICEs integrated in tandem. Therefore, it suggests that, similarly to IMEs, the prevalence of CIMEs is much larger than the one of ICEs in *S. salivarius*. The only other published search of CIMEs in a large array of genomes (303 of *Streptococcus agalactiae*) concerns CIMEs integrated in the 3′ end of the tRNALys CTT gene alone or in accretion with ICEs or IMEs [51]. It demonstrated the presence of 215 CIMEs deriving from ICEs and IMEs besides 88 ICEs and 66 IMEs integrated in this locus. Taken at whole, these previous results and the present work suggest that the prevalence of CIMEs is very high.

The detected ICEs, IMEs and CIMEs are expected to transfer by conjugation. This has been confirmed for ICE*St3*-related ICEs ([32,47] and for one IME [47]. Furthermore, apart from the case of identical or almost identical strains (such as Nu10 and Yu10), the comparison of the distribution patterns of related ICEs or IMEs and of the phylogenetic tree Figure 1 shows that these elements were horizontally transferred between strains. In this work, we searched for signature genes encoding integrase, CP, relaxase and VirB4 proteins but we did not search for other genes needed for conjugative transfer of ICEs nor for *trans* mobilization of IMEs. Therefore, some of the elements that are reported as ICEs or IMEs may actually be decayed ICEs that do not encode their own transfer or decayed IMEs unable to subvert conjugation apparatus of conjugative elements. However, most of the decayed ICEs probably keep their transfer ability by *trans*-mobilization by related conjugative elements. The best example is dICE_*Ssal39-01_fda* whose “conjugation” module differs from that of ICE*St3* by the precise deletion of the genes encoding the T4SS proteins but not of all other genes and sequences involved in transfer. Furthermore, decayed elements could be cis-mobilizable if they are integrated in tandem with a related or distantly related functional IME or ICE encoding an integrase able to recognize the *attL* and *attR* sites of the composite structure. *Cis*-mobilization of distantly related elements is likely rare since their *att* sites are generally very different. However, tandem structures can have excision patterns that can be somewhat surprising. For example, IME_*Sag2603_tRNAlys* from *S. agalactiae* is integrated in the 3′ end of a tRNALys gene in tandem with a dICE, generating the composite structure *attL*_ICE_-dICE-*attI*-IME-*attR*_IME_-3′ end of a tRNALys gene. Although these two integrated elements encode very different tyrosine integrases (<30% identity) and have very different *att* sites, the IME excises by site-specific recombination between the chimerical *attI* site and *attR*_IME_ and the whole composite element excises by site-specific recombination between *attL*_ICE_ and *attR*_IME_ [37].

ICEs and/or IMEs were found to be integrated specifically in 12 different target genes among which tRNALeu, tRNALys, *rpmG*, *rpsI* and *fda* are the most frequent. Integrations in tandem were observed only in three of these genes (*rpmG*, *rpsI* and *fda*). This suggests that IMEs that integrate specifically in the two frequently target genes encoding tRNALeu and tRNALys cannot integrate in the *att* sites flanking a resident element. However, it should be noticed that in *S. agalactiae*, IMEs related to the IMEs of *S. salivarius* that are specific of tRNALys genes are frequently integrated in tandem in tRNALys genes with ICEs distantly related to ICE*St3* and with CIMEs [56]. Although no tandem of ICEs has been identified in *S. salivarius*, tandems of ICEs belonging to different families were found in *Clostridium difficile* [57] and in *S. suis* [18]. In this work, we did not detect tandems of IMEs or ICEs belonging to the same family, as previously described in streptococcal genomes [16,17,18]. This could be due to an inhibition of conjugation or a surface exclusion by a resident element in the recipient strain as testified for various ICEs (for a review see [2]). It could also be due to an instability of tandems resulting from recombination or interactions between related elements, as shown for various ICEs including ICE*St3* [17,58,59].

We found that, in all composite regions, the most decayed elements, i.e., CIMEs, are always the most distant elements from the target genes. In the same way, the less decayed elements are located at the 3′ end of the target gene (*attR* end) in tandems integrated in the 3′ end of tRNALys genes from *S. agalactiae* [56] and in the 3′ end of the tmRNA genes of *Escherichia coli* and *Salmonella enterica* [60]. This structure probably results from the integration of an incoming element in the *attR* site of a decayed resident element that retains its *attL* and *attR* sites. We also found tandems of two functional unrelated elements (ICE and IME, or two IMEs). This structure probably results from the integration of an incoming element in the *attR* site flanking an unrelated functional resident element. Another scenario involving the acquisition of the whole composite element cannot be excluded. It was actually observed for composite structures including CIMEs and ICEs [2,61,62] or two unrelated ICEs [63]. Comparison of module compositions also suggests that recombination between related or unrelated ICEs, IMEs and CIMEs, likely integrated in tandem, plays a major role in the evolution and plasticity of ICEs and IMEs [2].

This work is one of the very few studies that identifies precisely the boundaries of a large number of various classes and families of integrated elements able to transfer by conjugation. All the integration specificities found in ICEs and IMEs from *S. salivarius* except one were previously described in other *Streptococci* [12,13,14,15,16] and their possible impact on the host fitness were previously discussed [3]. Globally, it seems that these integration specificities have evolved to reduce their impact on the host fitness to allow integration into a large array of strains and species and often to allow their mobilization *in cis* or *in trans* by other mobile elements ([47] for this last point). It appears that most elements encoding tyrosine recombinases integrate in the 3′ end of essential housekeeping genes and some in their 5′ end but without changing the sequence of the functional product (either tRNA or protein). By contrast, ICEs encoding serine integrases target internal sites of conserved genes encoding dispensable proteins. It was hypothesized that stimuli that induce the expression of the target gene also induce the excision of the integrated element and that the excised element controls its provisional maintenance by replication as an extrachromosomal element. Some ICEs target intergenic regions: (i) Tn*916*-related ICEs integrate preferentially in AT-rich short sequences found mainly in intergenic regions and/or in other mobile genetic elements and (ii) Tn*GBS2*-related elements encoding DDE transposases integrate 15 or 16 bp upstream from the −35 box of promoters recognized by sigma A [45], probably without modifying the expression of the downstream gene. IMEs (*n* = 11) encoding a serine recombinase related to the one of IME_*Sol3089_ND* described previously [16] are also integrated in various intergenic regions. Therefore, as for Tn*GBS2*-related elements, these elements seem to target transcription signals that belong either to promoters or terminators and are present in intergenic regions.

Therefore, the main impact on host fitness probably results from the expression of the cargo genes carried by the element. Some of these cargo genes may have dual properties being: (i) in certain circumstances, advantageous for the strain and therefore for the element or (ii) in other circumstances, advantageous for the element and consequently disadvantageous for the strain. For instance, five elements encode putative abortive infection systems (Abi) that would cause the death of cells infected by bacteriophages to prevent phage propagation. Therefore, Abi systems are advantageous for bacterial strains. However, recent studies have shown that various Abi systems are also toxin-antitoxins (TA) systems [64,65,66]. These systems kill the cells that lost the MGE that encodes them. They are also involved in the competition between incompatible conjugative elements and in their maintenance in bacterial population.

Furthermore, 28 elements encode RM systems that are advantageous for the cell since they confer resistance to bacteriophage. Most of them are type II RM systems, carried by ICE*St3* elements and located in a conserved position at the left of the regulation module. Since various type II RM systems are TA systems involved in plasmid maintenance [67,68], this suggests that type II RM are not only involved in bacteriophage resistance [69], but also in the selfish maintenance of ICE*St3*-related elements. Besides their advantageous function in strain competition, as for type II RM and TA systems, the production of bacteriocin that is encoded by four elements can also be viewed as an addiction system: harmless for cells harboring MGEs and harmful for cells that lost the elements.

Many elements from *S. salivarius* carry other cargo genes that have no role in the maintenance of the element in the cell but may increase bacterial fitness. Hence, 45 of them encode resistance to antimicrobial compounds. These include all ICEs, IMEs and MGIs that encode resistance to antibiotics. Resistance to tetracycline and erythromycin are carried mainly by the well-known Tn*916*-related elements. An MGI deriving from an ICE related to Tn*1549* (MGI_*SsaF4-20_rumA*) encodes a chloramphenicol acetyltransferase that we demonstrated to confer resistance to chloramphenicol [33]. Furthermore, two IMEs integrated in *oriT* of conjugative elements [47] encode a *lsa*(C) gene that is known to confer cross-resistance to lincosamides, streptogramin A and pleuromutilins in *S. agalactiae* [54]. In addition to these resistance genes, several ICEs and IMEs encode multidrug efflux transporter (MFS) that might also be involved in resistance to antibiotics. These data indicate that conjugative and mobilizable elements from *S. salivarius* are likely to be major determinants for the spreading of antibiotic resistance genes.

Moreover, 11 IMEs from *S. salivarius* encode *qacE* genes that are known to confer efflux-mediated resistance to QACs. QACs are disinfectants used in hospitals and food-processing environments to ensure microbiological safety [70]. QAC resistance genes are generally carried on plasmids and/or integrons. Here, we demonstrate that QAC genes are also carried by IMEs and are therefore likely to spread by mobilization. Several *S. salivarius* elements, mainly CIMEs, carry resistance to cadmium, a highly poisonous metal air pollutant [71]. Several elements from *S. salivarius* also encode proteins required for growth at 4 °C or involved in redox reactions or in the metabolism of amino acids, lipids or carbohydrates. For instance, Delorme et al. [31] described that *S. salivarius* K12 encodes all enzymes devoted to lactose utilization by the tagatose-6P pathway. Our analysis indicates that these enzymes are encoded by an MGI in this strain and that other elements (one dICE and two CIMEs) deriving from ICE*St3* also encode this metabolism. Hence, acquisition of such fitness mobile elements can be viewed as an important adaptive mechanism enabling survival of *S. salivarius* in a changing environment.

## Figures and Tables

**Table 1:** Diversity of the relaxases and CPs associated with serine and tyrosine integrases in IMEs/dIMEs.

Integrase Type (Number of IMEs/dIMEs)	Relaxase Families (Number of Relaxases)	CP Families (Number of CPs)	IME Superfamilies ^1^
Tyrosine (*n* = 155)	Rel_PF02486_2 (*n* = 65)	none (*n* = 65)	IME_PF02486 (IME_class_1)
	Rel _PF02486_4 (*n* = 4)	TcpA_2 (*n* = 4)	
	Rel _PF02486_5 (*n* = 21)	none (*n* = 22)	
	Rel _PF02486_6 (*n* = 22)	none (*n* = 22)	
	Rel _PF01719 (*n* = 28)	TcpA_12 (*n* = 28)	IME_PF01719 (IME_class_2)
	Rel_PF01719-PF0910_4 (*n* = 3) **Rel_PF01719-PF0910_5 (*n* = 4)**	TcpA_6 (*n* = 1) **TcpA_14** (*n* = 1) none (*n* = 1) TcpA_12 (*n* = 4)	IME_ PF01719-PF0910 (IME_class_4)
	Rel_PHA00330_1 (*n* = 3)	**TcpA_15** (*n* = 1) none (*n* = 2)	IME_PHA00330 (IME_class_3)
	Rel_PHA00330_2 (*n* = 2)	TcpA_8 (*n* = 2)	
	Rel_PHA00330_3 (*n* = 1)	**TcpA_13 (*n* = 1)**	
	Rel_PF13814 (*n* = 1)	none (*n* = 1)	IME_PF13814 (IME_class_8)
Serine (*n* = 12)	Rel_PF13814 (*n* = 12)	VirD4 (*n* = 12)	

In bold, new families of relaxases and CPs. ^1^ The IME superfamily name comprises the pfam accession number of the relaxase catalytic domain(s). The correspondence with the names given in [16] is indicated in brackets.

**Table 2:** Composite structures resulting from tandem integration.

Structure of Composite Regions	Prevalence
CIME-IME-target gene	7
microCIME-ICE-target gene	6
CIME-ICE-target gene	4
IME-IME-target gene	3
CIME-IME-target gene	3
microCIME-IME-target gene	2
2 microCIMEs-dICE-target gene	2
IME-ICE-target gene	2
ICE-IME-target gene	2
CIME-IME-IME-target gene	2
microCIME-CIME-ICE-target gene	1
CIME-dICE-target gene	1
CIME-ICE-IME-target gene	1
MGI-IME-target gene	1

## Supplementary Materials

The following are available online at https://www.mdpi.com/2073-4425/11/9/999/s1, Table S1: Date and origin of *Streptococcus salivarius* sampling and genbank accession number. Table S2: Coordinates of *Streptococcus salivarius* integrative elements transferable by conjugation. Table S3: Diversity and abundance of *Streptococcus salivarius* integrative elements transferable by conjugation. Figure S1: Schematic representation of ICEs, IMEs, MGIs and CIMEs. Figure S2: Function of the cargo genes carried by the conjugative elements integrated in the genomes of *S. salivarius*. File S1: nucleotidic sequences of elements.
