# Supplementary material for: Abundance, Diversity and Role of ICEs and IMEs in the Adaptation of Streptococcus salivarius to the Environment
Source: Genes (Basel). 2020 Aug 26;11(9):999. doi: 10.3390/genes11090999 (PMC7563491; doi:10.3390/genes11090999)
Supplement: Supplementary file 1 [file genes-11-00999-s001.zip › Supplementary files/Lao_Fig S2.pdf]

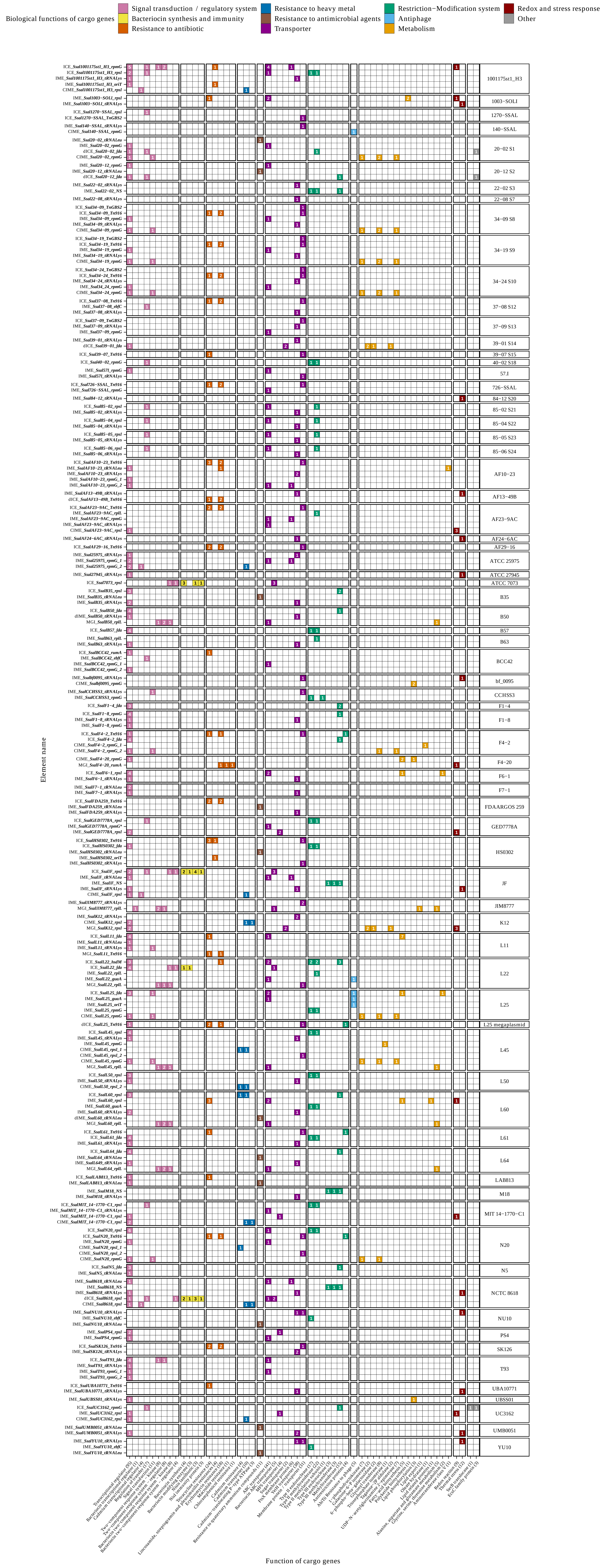

Figure S2: Function of the cargo genes carried by the conjugative elements integrated in the genomes of ***S. salivarius***. The numbers correspond to the number of genes for each function.
